# *In vivo *site-specific biotinylation of proteins within the secretory pathway using a single vector system

**DOI:** 10.1186/1472-6750-8-41

**Published:** 2008-04-18

**Authors:** Andrea Predonzani, Francesca Arnoldi, Alejandro López-Requena, Oscar R Burrone

**Affiliations:** 1 Molecular Immunology Group, International Centre for Genetic Engineering and Biotechnology, Padriciano 99, 34012 Trieste, Italy; 2 Department of Antibody Engineering, Center of Molecular Immunology, P.O. Box 16040, Havana 11600, Cuba

## Abstract

**Background:**

Due to its extremely high strength, the interaction between biotin and (strept)avidin has been exploited for a large number of biotechnological applications. Site-specific biotinylation of proteins *in vivo *can be achieved by co-expressing in mammalian cells the protein of interest fused to a 15 amino acid long Biotin Acceptor Peptide (BAP) and the bacterial biotin-protein ligase BirA, which specifically recognizes and attaches a biotin to the single lysine residue of the BAP sequence. However, this system is mainly based on the contemporaneous use of two different plasmids or on induction of expression of two proteins through an IRES-driven mechanism.

**Results:**

We developed a single bigenic plasmid that contains two independent transcriptional units for the co-expression of both the protein tagged with BAP and an engineered version of the BirA enzyme. Upstream of the cDNA encoding BirA, a signal secretion leader sequence was added to allow translocation of the enzyme to the secretory pathway. Three different recombinant antibodies in the scFv format, a membrane bound and secretory truncated IgE Fc fragment and a soluble version of the human IgE high affinity receptor were shown to be efficiently biotinylated and to maintain their binding properties in immunofluorescence microscopy, flow cytometry and ELISA assays.

**Conclusion:**

The present study shows the universal applicability to both secretory and membrane bound proteins of a single bigenic plasmid to induce the site-specific *in vivo *biotinylation of target molecules tagged with a short acceptor peptide. These molecules could be easily obtained from supernatants or extracts of mammalian cells and used for a wide range of biological applications.

## Background

Biotin or vitamin H is a small hydrosoluble molecule produced by plants and a large number of prokaryotic organisms. It is involved in several metabolic pathways, including gluconeogenesis or fatty acid and amino acid catabolism, as a carboxyl carrier covalently linked to a specific lysine residue of a group of decarboxylases. This residue is located in a subunit called biotin carboxyl carrier protein (BCCP), and biotin is attached in an ATP-dependent reaction by a highly conserved family of biotin-protein ligases, the most characterized of which is the BirA enzyme from *E. coli *[1-4].

In addition to its physiological relevance, biotin has the interesting ability to bind to two homologous proteins: avidin, from the avian egg, and streptavidin, from bacteria. This interaction is the strongest non-covalent binding known (Kd = 10^-15 ^M) [5]. Taking advantage of this property, the interaction biotin-(strept)avidin has been exploited for a large number of biotechnological applications [6,7]. Currently, biotinylation of proteins is achieved by different chemical reactions. The major disadvantage of this approach is the uncontrollable binding of biotin to sites of the target protein that can be important for its biological activity [8,9].

To overcome this limitation, new approaches that mimic the physiological biotinylation of proteins have been developed, using different peptides containing an enzymatic biotinylation site [10-12]. One of the most useful and widely used is a 15 amino acid long peptide (biotin acceptor peptide or BAP) [13,14] that constitutes a specific and efficient target for the biotin-protein ligase BirA of *E. coli *[15], which attaches covalently one biotin molecule to the single lysine residue within the BAP sequence. When the BAP peptide is genetically fused to the protein of interest, it is possible to obtain the protein biotinylated through two alternatives: *in vitro*, using the tagged protein and the purified enzyme [16], or *in vivo *in mammalian cells following co-transfection with plasmids encoding both proteins [15,17,18]. The *in vivo *approach has the advantage of producing the tagged protein and the BirA enzyme at constant rates avoiding purification of both molecules.

Here we show the universal applicability to both secretory and membrane bound proteins of immunological interest of a biotinylation system based on the co-expression of a target protein and an engineered version of BirA enzyme encoded by a single bigenic plasmid. Secretory recombinant antibody derived molecules, for instance in the popular scFv format, a membrane bound and secretory IgE Fc fragment or a soluble version of a membrane receptor were shown to be efficiently biotinylated *in vivo*, offering a wide range of biological applications.

## Methods

### Construction of plasmids

The coding sequence for the BirA enzyme [GenBank: P06709] was amplified by PCR from genomic DNA of *E. coli *K12 strain with oligonucleotides: 5'-TGTGTGCACTCGATGAAGGATAACACCGTGCCA-3' (forward) and 5'-AGACTCGAGTTATTTTTCTGCACTACGCAGGGA-3' (reverse). The amplified fragment was ligated into *Apa*LI and *Xho*I sites of pUT-sec vector [19] to introduce a secretion signal. The *Hin*dIII/*Xho*I fragment from this plasmid was ligated into pcDNA3 vector (Invitrogen, Paisley, UK), obtaining the pcDNA3-sec-BirA plasmid.

The BAP sequence was obtained by annealing and elongating with DNA polymerase I Klenow large fragment (New England Biolabs, Beverly, MA) the two oligonucleotides: 5'-AGCTGGATCCGCCGGAGGCTCTGGAGGCCTGAACGATATTTCCGAAGCTCAGAAAAT-3' and 5'-ATCGAATTCTTAAGAGCCTTCGTGCCATTCGATTTTCTGAGCTTCGAAAAT-3'. The resulting fragment encodes a 7 amino acid linker (GSAGGSG) between the protein of interest and the BAP sequence GLNDIFEAQKIEWHE. This fragment was inserted into a pcDNA3 vector coding for the SV5 protein tag [20,21], downstream SV5 between restriction sites *Bam*HI/*Eco*RI.

Plasmids coding for scFv^P3 ^and scFv^1E10 ^have been previously described [22,23]. These plasmids were digested with *Hin*dIII and *Bsp*EI restriction enzymes to introduce the scFv*s *upstream the SV5-BAP sequence, obtaining the pcDNA3-scFv-SV5-BAP plasmids. Every time scFv*s *of different specificities were required, the *Hin*dIII/*Bsp*EI fragments encoding the scFv*s *of interest were substituted to the previous ones.

To clone the transcriptional unit of the BirA gene into the plasmid codifying for the scFv-SV5-BAP protein, the *Bgl*II/*Bbs*I fragment derived from pcDNA3-sec-BirA was blunted with Klenow enzyme and inserted into the *Nru*I-digested scFv-SV5-BAP plasmids, resulting in the bigenic scFv-SV5-BAP-BirA plasmids.

To construct the bigenic αD1D2-SV5-BAP-BirA plasmid (αD1D2), the *Nde*I/*Bsp*EI fragment derived from pcDNA3-sdα [24] was ligated with the scFv-SV5-BAP-BirA vector, previously digested with the *Nde*I and *Bsp*EI restriction enzymes to remove the scFv.

The sequence coding for the truncated form of IgE antibody t-IgE with a SV5 tag (SV5-εC_H_3C_H_4) was previously obtained [25]. The BAP sequence was inserted at the C-terminus of the soluble form (t-sIgE) between *Bam*HI and *Eco*RI sites. For the membrane-bound format t-m_L_IgE, with the transmembrane domain (TM) at the C-terminus, both SV5 and BAP tags were cloned at the N-terminus between *Apa*LI and *Bst*BI sites, after amplifying the fragment SV5-BAP from the template scFv^1E10^-SV5-BAP with oligonucleotides: 5'-TCTGTGCACTCGGAAGGCAAACCAATCCCAAAC-3' and 5'-TCTTTCGAATCTGCACAAGAGCCTTCGTGCCATTC-3'. The bigenic SV5-εC_H_3C_H_4-BAP-BirA (t-sIgE) and SV5-BAP-εC_H_3C_H_4-TM-BirA (t-m_L_IgE) plasmids were obtained as described above for scFv*s*.

**Figure 1:**
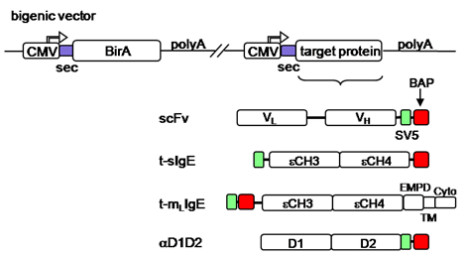
**Representation of the bigenic vector and gene constructs**. Scheme of the different model target protein constructs used. EMPD, TM and Cyto correspond, respectively, to the extracellular membrane proximal, transmembrane and cytoplasmic domains of membrane bound t-IgE; green box, 11 amino acid long SV5 tag; red box, 15 amino acid long biotin acceptor peptide BAP; sec, secretory signal peptide.

All constructs are represented in Fig. 1.

### Cell culture, transfection and selection

HEK293 and HEK293T/17 cells from American Type Culture Collection (Rockville, MD, numbers CRL-1573 and CRL-11268, respectively) were cultured in Dulbecco's modified Eagle's medium (DMEM, Gibco, Paisley, UK) supplemented with 10% heat inactivated fetal calf serum (FCS) (Gibco), 50 μg/ml gentamycin (Gibco) and 2 mM L-glutamine.

Transient transfections of HEK293T/17 cells and stable transfections of HEK293 cells were performed essentially as described by Sambrook *et al*. [26], using circular or linearized plasmids respectively. To select stable clones, 0,4 mg/ml Geneticin (G418, Gibco) was added to the medium 24 h after transfection. Selected clones were screened for their production of biotinylated proteins by ELISA assay.

RBL-SX38 cells expressing the high-affinity receptor for IgE (FcεRI) [27] were cultured in DMEM supplemented with 10% heat inactivated FCS, 50 μg/ml gentamycin, 2 mM L-glutamine, 0,8 mg/ml Geneticin and 3,5 mg/ml glucose.

The Sp2/0 stable transfectoma displaying the 1E10 idiotype in the membrane small immune protein (membrane SIP) format [28] was previously described [22]. Cells were cultured in RPMI 1640 medium (Gibco) supplemented with 10% heat inactivated FCS, 50 μg/ml gentamycin, 2 mM L-glutamine, 1 mM sodium piruvate and 0,4 mg/ml Geneticin.

The A20 stable transfectoma expressing the full human membrane IgE was previously obtained [28] and cultured as the Sp2/0 transfectoma above described.

### Preparation of samples

Supernatants from transiently transfected cells were collected 48 h post transfection. 24 h before collection, the culture medium was replaced with a serum-free medium supplemented with 100 μM biotin (Fluka). To remove free biotin, after collection supernatants were extensively dialyzed against PBS.

In the case of stable clones, supernatants were collected after 24 h of culture in serum-free medium supplemented with biotin and dialyzed.

Cellular extracts were prepared in TNN lysis buffer (100 mM Tris-HCl, pH 8, 250 mM NaCl, 0.5% NP-40) at 4°C, supplemented with a Protease Inhibitor Cocktail (PIC, Sigma) according to manufacturer's instructions, and were centrifuged at 15,800 × *g *for 5 min.

### Enzyme-linked immunoadsorbent assay (ELISA)

Nunc Immune-Plates Maxi Sorp (Nunc, Denmark) were coated with the specific ligands in carbonate buffer: 10 μg/ml chimeric 1E10 and P3 antibodies [29] for scFv^P3 ^and scFv^1E10 ^respectively; 10 μg/ml anti-human IgE antibody (DAKO) for t-sIgE; 10 μg/ml purified human IgE (Calbiochem, Canada) for αD1D2; 1 μg/ml streptavidin (Invitrogen) for biotinylated scFv^9E1^. After 1 h, plates were washed with PBS-Tween20 0,05% and blocked with PBS-Tween20 0,05%-BSA (bovine serum albumin) 1%. Interactions were detected using a mouse anti-SV5 monoclonal antibody (mAb) (Invitrogen) and, as a secondary antibody, a horseradish peroxidase (HRP)-conjugated anti-mouse IgG (γ-chain specific) antibody (Pierce, Rockford, IL), whereas biotinylation of molecules was checked by using HRP-conjugated streptavidin (Amersham). All antibodies were diluted according to the manufacturer's instructions. Reaction was developed with the 3,3',5,5'-tetramethylbenzidine (TMB) reagent (Sigma) and read at 450 nm with a Microplate 550 reader (BioRad, Hercules, CA).

### SDS-PAGE and Western blot

Samples were analyzed by 10% SDS-PAGE under reducing conditions in Tris-glycine buffer. Following electrophoresis, gels were blotted onto PVDF membrane (Millipore) with standard conditions [30]. Membranes were blocked with PBS-milk 5% and subsequently incubated with specific antibodies.

To estimate the relative amount of biotinylated versus non-biotinylated proteins, a gel retardation assay was used. Samples (dialyzed supernatants or cell extracts) were boiled for 5 min, incubated for 1 h at 4°C with 1 μg of purified streptavidin (Invitrogen) and then resolved by SDS-PAGE under reducing conditions. The resulting complexes were detected by Western blot using an anti-SV5 mAb (Invitrogen) and an HRP-conjugated anti-mouse IgG (γ-chain specific) antibody (KPL, Kirkegaard and Perry Laboratories, Gaithersburg, MD), diluted according to the manufacturer's instructions.

To detect the cytosolic protein NSP5, a guinea pig anti-NSP5 anti-serum [31] and an HRP-conjugated anti-guinea pig IgG antibody (KPL) were used.

### Flow cytometry assays

The Sp2/0 stable transfectoma expressing the 1E10 membrane SIP was incubated with undiluted dialyzed supernatants from scFv^P3^-expressing transfectoma for 1 h at 4°C, washed twice with PBS-BSA 3%, and incubated again with streptavidin-Quantum Dots 655 (Invitrogen), diluted 1:1,000 in PBS-BSA 3%. Streptavidin binding was detected by FACS with a FACSCalibur flow cytometer (Beckton Dickinson, Mountain View, CA) and data were analyzed with the CellQuest software. As negative controls, supernatants containing scFv^1E10 ^or the non-biotinylated scFv^P3 ^were used.

Similarly, RBL-SX38 cells were incubated with supernatants from scFv^9E1 ^or t-sIgE-expressing transfectomas. As negative controls, supernatants with the non-biotinylated proteins were used.

Cells expressing transiently the t-m_L_IgE protein were washed twice with PBS and once with PBS-BSA 3% and then incubated with streptavidin-Quantum Dots 655 or a fluorescein isothiocyanate (FITC)-conjugated goat anti-human IgE (ε-chain specific) antibody (KPL) (diluted according to the manufacturer's instructions).

### Immunofluorescence microscopy

Sp2/0 and A20 transfectomas and RBL-SX38 cells were plated on poly-L-lysine (Sigma) coated glass coverslips for 15 min, fixed with 3,7% paraformaldehyde for 10 min and then incubated with supernatants containing the specific ligand for 1 h at room temperature, washed and incubated with streptavidin-Quantum Dots for 1 h. Samples were analyzed by confocal microscopy (Axiovert, Carl Zeiss). As positive controls, FITC-conjugated goat anti-human IgE (ε-chain specific) antibody (KPL), recognizing the self-dimerizing εC_H_4 domain of the membrane SIP, or the 9E1 mAb followed by a FITC-conjugated goat anti-mouse IgG (γ-chain specific) antibody (KPL) were used. Antibodies were diluted as for flow cytometry assays.

### Purification of biotinylated molecules

Biotinylated molecules from dialyzed supernatants were purified using SoftLink™ Soft Release Avidin Resin (V201B, Promega, Madison, WI, USA) according to the manufacturer's instructions. Briefly, an appropriate bed volume of resin was poured into an empty chromatography column (BioRad), washed, saturated with 5 mM biotin and regenerated. After equilibration with modified TBS buffer (50 mM Tris, 150 mM NaCl, pH 7.9), the supernatant was loaded and elution performed with 5 mM biotin in equilibration buffer. Fractions of 250 μl were collected and assayed by Western blot analysis. All steps were performed at room temperature.

## Results

To achieve site-specific biotinylation of proteins within the secretory pathway, two requirements must be fulfilled: i) the protein of interest tagged with the appropriate BAP sequence, and ii) the biotin protein ligase expressed within the secretory compartment, i.e. the endoplasmic reticulum (ER).

In all our constructs we have used the BAP sequence GLNDIFEAQKIEWHE, fused to either the N- or the C-terminus of the protein. The cDNA of the BirA enzyme was engineered by adding at the 5' end a sequence encoding a secretion signal leader peptide [19] to allow translocation of the enzyme into the ER (sec-BirA). For *in vivo *biotinylation of proteins within the secretory pathway in mammalian cells, we initially co-transfected two plasmids encoding the tagged protein of interest and the sec-BirA enzyme. However, since the concomitant expression from two different plasmids was sometimes not well achieved (in particular when selecting stable transfectomas), we constructed a single bigenic plasmid with two different and independent gene cassettes, one for the sec-BirA enzyme and the other for the target protein. The two cassettes were assembled in the same and the opposite transcriptional orientation. Since the relative orientation of the two transcriptional units was irrelevant to the expression and the biotinylation levels of the BAP-tagged protein (not shown), we arbitrarily chose the vector with the two cassettes in the same orientation for all the different molecules tested (listed in Fig. 1).

**Figure 2:**
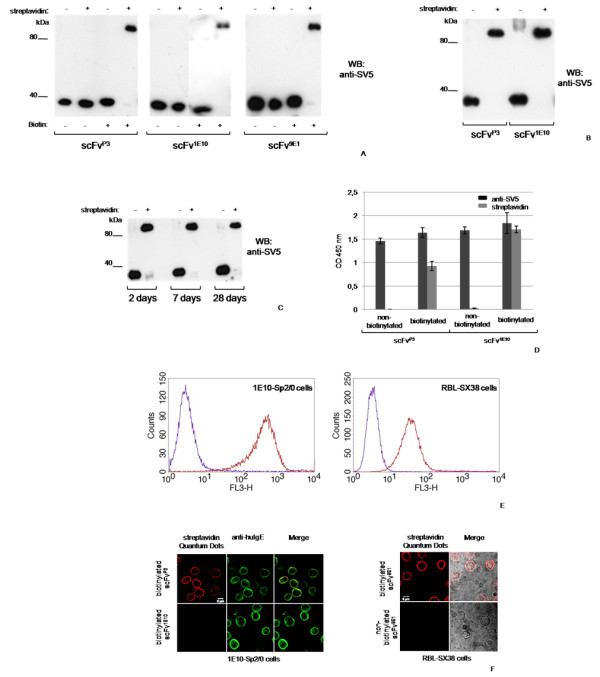
**Biotinylation of secretory scFv proteins**. Western blot gel retardation assay of supernatants from cells transiently transfected (A) or stably transfected (B) with the bigenic vector expressing the indicated scFv. (C) Supernatants from stably transfected cells taken at different culture times. Where indicated, cell cultures were supplemented with biotin for 24 h and dialyzed samples reacted with streptavidin. (D) ELISA of biotinylated or non-biotinylated scFv^P3 ^(coating chimeric mAb 1E10) and scFv^1E10 ^(coating chimeric mAb P3); binding was revealed with anti-SV5 tag mAb or HRP-conjugated-streptavidin. (E) Flow cytometry and (F) immunofluorescence microscopy of biotinylated scFv*s *on cells expressing specific cell-surface ligands. scFv^P3 ^(left panels) was reacted with cells displaying idiotype 1E10; scFv^9E1 ^(right panels) was reacted with RBL-SX38 expressing human FcεRI. Binding was performed with biotinylated (red) or non-biotinylated (violet) scFv*s *and with Quantum Dots-conjugated streptavidin. The green signal corresponds to FITC-conjugated anti-human IgE (ε-chain specific) antibody that recognizes the ε-membrane SIP displaying the idiotype.

We first tested our bigenic plasmid system with three different model scFv*s *expressed as secretory proteins that were derived from: i) mAb P3, specific for *N*-glycolyl-containing gangliosides [32], ii) mAb 1E10, an anti-idiotype antibody specific for mAb P3 [33] and iii) mAb 9E1, specific for the α-chain of the human IgE high affinity receptor (FcεRI) [24]. Culture supernatants from transiently transfected HEK293T/17 cells cultured in the presence of biotin, were first dialyzed to eliminate free biotin and then analyzed by Western blot (WB) in a gel retardation assay in the presence of streptavidin. The complex formed by streptavidin and biotinylated protein resists SDS-PAGE conditions and is retarded in relation to the non-biotinylated molecules that do not bind streptavidin. As shown in Fig. 2A, almost all molecules of the three different scFv*s *were biotinylated, indicating the high efficiency of the method. For two scFv*s*, similar analysis was then repeated with supernatants from stably transfected HEK293 cells showing similar biotinylation efficiency (Fig. 2B). In addition, both the expression (around 3–6 μg/ml, data not shown) and the biotinylation levels of molecules remained almost identical over time, as shown with samples taken after 2, 7 or 28 days of culture of the transfectoma expressing the scFv^1E10 ^(Fig. 2C). No signs of increased cell death were observed for the different transfectomas, indicating that the expression of BirA in the ER is not toxic for cells.

The binding properties of the biotinylated scFv*s *were assayed by ELISA, and for two of them also by flow cytometry and immunofluorescence assays (Fig. 2D–F), using cells displaying on their membranes the respective ligands. scFv^P3 ^was tested on cells displaying the 1E10 idiotype, whereas the scFv^9E1 ^was tested on RBL-SX38 cells that express the high affinity receptor FcεRI. In both cases, a clear and ligand-specific binding of the biotinylated molecules was demonstrated using streptavidin coupled to Quantum Dots, which provided a stronger signal compared to streptavidin conjugated with traditional fluorophores (not shown).

**Figure 3:**
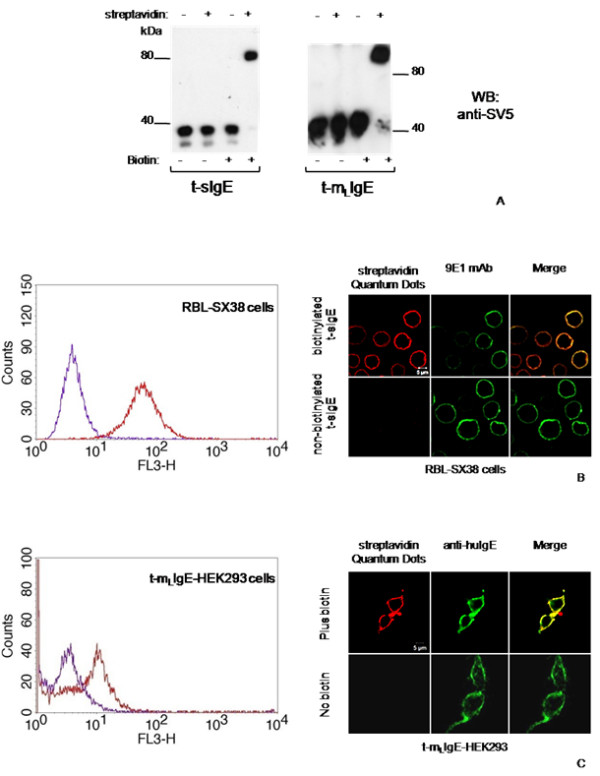
**Biotinylation efficiency of secretory and membrane bound t-IgE**. (A) Western blot gel retardation assay of supernatants (t-sIgE) or cell extracts (t-m_L_IgE) from transiently transfected cells expressing secretory or membrane bound t-IgE. Where indicated, cell cultures were supplemented with biotin for 24 h and supernatants (dialyzed) or cell extracts reacted with streptavidin. (B) Flow cytometry and immunofluorescence microscopy assay on RBL-SX38 cells expressing human FcεRI incubated with biotinylated (red) or non-biotinylated (violet) t-sIgE; mAb 9E1 was detected with FITC-conjugated anti-mouse IgG antibody. (C) Flow cytometry and immunofluorescence microscopy assay of t-m_L_IgE displayed on transiently transfected HEK293T/17 cells, cultured in the presence (red) or absence (green) of biotin.

We also co-expressed with sec-BirA a recombinant truncated version of the human IgE heavy chain protein either in its secretory or membrane bound form [25], to investigate and compare the efficiency of *in vivo *biotinylation of the same target protein in the two different versions. Retardation assays of the secretory (t-sIgE) and membrane bound (t-m_L_IgE) molecules revealed that also in this case most of them were biotinylated (Fig. 3A). No differences in biotinylation levels were observed when the BAP sequence was fused to the N- or the C-terminus of t-IgE: indeed, in both forms the same ER luminal sequence is present and they diverge in the position of the BAP tag. The binding activity of the soluble biotinylated t-sIgE was not compromised by biotinylation, as its efficient binding to the high affinity FcεRI on RBL-SX38 cells was clearly detected with streptavidin-Quantum Dots, both by flow cytometry and immunofluorescence microscopy (Fig. 3B). Similarly, the membrane form t-m_L_IgE was effectively displayed on the cell surface and efficiently biotinylated, as revealed by fluorescent streptavidin binding (Fig. 3C).

**Figure 4:**
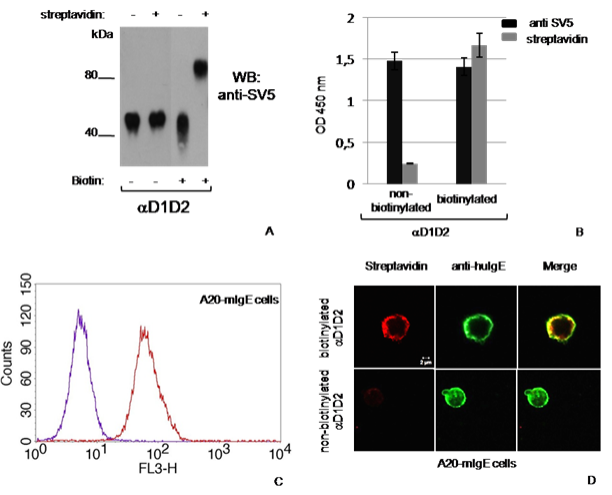
**Biotinylation of the soluble high affinity receptor FcεRI α-chain**. A) Western blot gel retardation assay of culture supernatants from cells expressing αD1D2. Where indicated, cell cultures were supplemented with biotin for 24 h and dialyzed samples reacted with streptavidin. (B) ELISA on IgE-coated plates of αD1D2, revealed with anti-SV5 tag mAb or HRP-conjugated streptavidin. (C) Flow cytometry and immunofluorescence microscopy assay with biotinylated (red) or non-biotinylated (violet) αD1D2 on transfected A20 cells expressing human membrane IgE; the green signal corresponds to FITC-conjugated anti-human IgE (ε-chain specific) antibody.

Finally, we also expressed the BAP-tagged soluble version of the FcεRI α-chain (αD1D2) [24], which became highly biotinylated and retained its IgE binding activity (Fig. 4).

**Figure 5:**
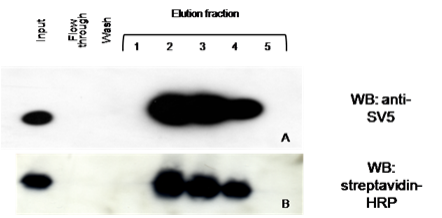
**Purification of biotinylated scFv from culture supernatant**. Western blot analysis of biotinylated scFv^P3 ^purified in a low affinity avidin column and eluted with biotin, detected with anti-SV5 tag mAb (A) or with HRP-conjugated streptavidin (B). The eluted molecules (more than 90% of input) were about 10-fold more concentrated than in the supernatant.

We tested the feasibility of purification of one of the *in vivo *biotinylated scFv*s*, using a commercially available modified avidin with low binding affinity for biotin. As shown in figure 5, the biotinylated scFv was eluted with high yield and purity, and ten times concentrated with respect to the culture supernatant.

**Figure 6:**
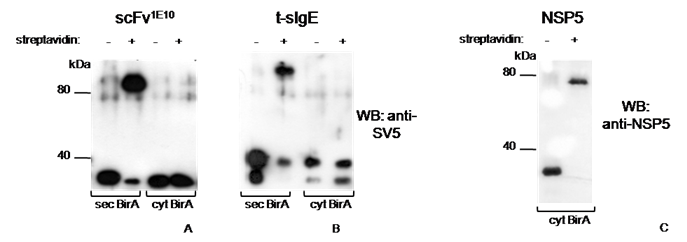
**Biotinylation dependence on BirA localization**. Western blot gel retardation assay on cells co-transfected with plasmid constructs encoding secretory (sec) or cytosolic (cyt) BirA, and scFv^1E10 ^(A), t-sIgE (B) or NSP5 (C). Where indicated, supernatants (dialyzed) or cell extracts were reacted with streptavidin.

As shown above, the ectopic localization in the ER of the BirA enzyme did not compromise its enzymatic activity, allowing biotinylation of both secretory and membrane proteins. As expected, biotinylation was dependent on the presence of the enzyme BirA in the right compartment. In fact, only when the secreted scFv^1E10 ^or t-sIgE were co-expressed with sec-BirA, they became almost completely biotinylated, whereas no biotinylation took place either when scFv^1E10 ^and t-sIgE were expressed alone or co-expressed with cytosolic BirA (i.e: with no secretory signal peptide). On the other hand, cytosolic BirA was competent, as expected, in biotinylating a cytoplasmic localized protein, the rotavirus non-structural protein NSP5 [34] (Fig. 6).

## Discussion

The biotin-avidin/streptavidin binding is the strongest non-covalent interaction known in nature. This property is exploited as a biological tool for a wide range of applications, like purification or immunodetection of target molecules to which biotin is covalently attached. Most approaches for biotinylation of molecules are based on chemical procedures, which have as a major disadvantage the lack of specifity for the biotin incorporation site. Moreover, coupling reactions could modify residues that affect the three-dimensional conformation or the specific properties of the target molecule [8,9]. To overcome these limitations, the enzymatic biotinylation was introduced [35]. This technology was improved by the discovery of new sequence specific substrates for biotinylation enzymes that could be fused to the target molecules. Some of these sequences are more than 100 amino acid long [12], but shorter ones are also available. Among the latters, a very convenient one is the 15 amino acid long biotin acceptor peptide (BAP), originally described by Beckett et al. [14], which contains a single lysine as a biotin acceptor residue. Biotinylation can be obtained *in vitro *using proteins tagged with BAP and incubated with the purified enzyme [6,36,37]; alternatively, a similar strategy can be used *in vivo *by co-expressing the target protein and the biotin-protein ligase BirA. To co-express them in mammalian cells, either a two plasmid co-transfection system has been used [15,18,38] or a bicistronic plasmid based on the IRES sequence [39]. For this strategy to be successful with proteins within the secretory pathway, the BirA enzyme, which is a cytosolic protein, must be engineered in a way that allows its translocation to the ER. We achieved such translocation using a validated secretory signal leader peptide from a mouse immunoglobulin heavy chain [19]. This was essential since the cytosolic version of BirA did not sustain biotinylation of secreted proteins, while it did so very efficiently for cytosolic proteins like rotavirus NSP5. Although a two-plasmid system can be used, we arranged our system in a single bigenic plasmid, which was particularly useful for the selection of stable transfectomas co-expressing both proteins, and able to sustain high levels of biotinylation. In addition, cell viability was not compromised, allowing the continuous production of the biotinylated molecule of interest.

Here we have shown the effective *in vivo *biotinylation of different recombinant molecules of immunological interest. However, this strategy can be applied to any other protein within the secretory pathway. The simple method we used to induce BirA-driven high efficient biotinylation was effective for a number of different secretory and membrane bound proteins, with the BAP tag positioned both at the C- and N-terminus. Fusion of the BAP peptide neither caused intracellular accumulation nor compromised secretion of the scFv*s *(data not shown). We have also observed biotinylation of other proteins with BAP inserted in defined structural internal loops (Arnoldi F. and Burrone O.R., unpublished).

In order to get biotinylation strictly dependent on exogenously added biotin, cells must be cultured in biotin-free medium and dialyzed serum. This is an attractive aspect of the technology, because it allows performing biotin pulse-labelling, as well as production of non-biotinylated BAP-tagged proteins.

## Conclusion

This methodology provides many possibilities of application. Biotinylated molecules obtained by transfection of cells with the single bigenic plasmid can be simply recovered and easily purified from the culture supernatants or cell lysates using commercially available reagents, like beads with covalently immobilised monomeric avidin, from which bound proteins can be eluted using free biotin. Furthermore, biotinylated membrane bound proteins can be easily detected on the surface of cells with streptavidin conjugated with different fluorophores, including Quantum Dots (which showed higher efficiency). Moreover, culture supernatants containing biotinylated secreted proteins can be directly used without further purification in a number of different immunochemical techniques, such as Western blot, ELISA, immunofluorescence microscopy and flow cytometry.

## Authors' contributions

AP performed the construction of bigenic vectors, data acquisition, Western blot and binding assays. FA was responsible for engineering and construction of BirA plasmids setting the conditions of the shift assay and data acquisition. ALR performed the modification of scFv^P3 ^and scFv^1E10 ^vectors and provided scientific support. OB conceived, designed, and coordinated the original project and provided scientific support. All authors participated in writing the manuscript, and read and approved it in its final version.
